# Efficacy of Myopia Prevention in At-Risk Children: A Systematic Review and Network Meta-Analysis

**DOI:** 10.3390/jcm14051665

**Published:** 2025-02-28

**Authors:** Ssu-Hsien Lee, Bor-Yuan Tseng, Jen-Hung Wang, Cheng-Jen Chiu

**Affiliations:** 1 School of Medicine, Tzu Chi University, Hualien 970, Taiwan; 107311120@gms.tcu.edu.tw (S.-H.L.); 107311121@gms.tcu.edu.tw (B.-Y.T.); 2 Department of Medical Research, Buddhist Tzu Chi General Hospital, Hualien 970, Taiwan; paulwang@tzuchi.com.tw; 3 Department of Ophthalmology and Visual Science, Tzu Chi University, Hualien 970, Taiwan; 4 Department of Ophthalmology, Hualien Tzu Chi Hospital, The Buddhist Tzu Chi Medical Foundation, 707, Section 3, Zhongyang Rd, Hualien 970, Taiwan

**Keywords:** myopia prevention, atropine, red light, outdoor activities, systematic review, network meta-analysis

## Abstract

**Objectives:** To evaluate the efficacy of myopia prevention methods in children without pre-existing myopia. **Methods:** A network meta-analysis was conducted following the PRISMA-NMA guidelines. Comprehensive searches were performed in PubMed, Embase, and Cochrane CENTRAL databases. The analysis focused on randomized controlled trials evaluating myopia prevention strategies in children without prior myopia. Primary outcomes included annual changes in refraction and axial length, while secondary outcomes encompassed myopia incidence and adverse events. Effect sizes were reported as risk ratios (RR) or mean differences (MD) with 95% confidence intervals (CIs). Data synthesis utilized a random-effects model under a frequentist framework, with intervention efficacy ranked by P-scores. Study quality was assessed using the Cochrane risk-of-bias tool, and robustness was ensured via sensitivity and consistency analyses. **Results:** Low-level red light therapy and low-dose atropine were the most effective interventions for reducing refractive progression (MD: 0.48 D, 95% CI: 0.38–0.59 D; MD: 0.33 D, 95% CI: 0.23–0.43 D) and axial elongation (MD: −0.23 mm, 95% CI: −0.27 to −0.19 mm; MD: −0.12 mm, 95% CI: −0.16 to −0.08 mm). In addition, both significantly lowered myopia incidence (RR: 0.59, 95% CI: 0.45–0.79; RR: 0.55, 95% CI: 0.41–0.75). Outdoor activities and myopia awareness programs demonstrated moderate efficacy. Adverse events, including photophobia and dry eyes, were minor and self-limiting, with no serious complications reported. **Conclusions:** Low-level red light therapy and low-dose atropine are the most effective, generally safe strategies for preventing myopia in at-risk children without myopia, while a non-invasive approach, outdoor activities, provides moderate benefits.

## 1. Introduction

The global epidemic of myopia is a significant public health challenge, with prevalence rates reaching alarming levels worldwide [1], particularly in East and Southeast Asia, with nearly 80–90% of adolescents affected [2]. The increasing incidence of myopia, especially among children and adolescents, has raised concerns due to associated long-term risks [3], such as myopic maculopathy [4], cataracts [5], and glaucoma [6]. These potential complications underscore the necessity for early preventive interventions that focus not only on slowing myopia progression but also on addressing the condition before its onset [7].

Research on myopia prevention has traditionally concentrated on interventions for individuals already diagnosed with myopia [8]. Prevention strategies such as lifestyle modifications and pharmacological interventions [9], including increasing outdoor exposure, promoting physical activity, and, more recently, use of pharmacological agents such as low-dose atropine [10] and innovative light-based therapies like low-level red light treatment have been proposed [11]. While lifestyle interventions, such as outdoor activities, have limited efficacy in reducing myopia progression among myopic children, these approaches may help prevent the onset of myopia in children without the condition [12,13]. However, evidence remains fragmented, with varying results across different intervention modalities and populations [14,15,16], highlighting the need for a comprehensive network meta-analysis (NMA) specifically addressing children without myopia. This gap limits our understanding of the relative effectiveness and potential risks associated with different preventive methods for this population. To address this limitation, this systematic review and NMA of available interventions aimed to provide robust evidence to guide clinical practice and inform future research.

## 2. Materials and Methods

### 2.1. Study Design

This NMA aimed to evaluate the comparative efficacy of various interventions for preventing myopia in children without pre-existing myopia. The study adhered to the Preferred Reporting Items for Systematic Reviews and Meta-Analyses for Network Meta-Analyses (PRISMA-NMA) guidelines [17]. To ensure transparency and methodological rigor, the methodology was pre-specified and registered in the PROSPERO database (Registration No. CRD42024611052).

### 2.2. Eligibility Criteria

Studies meeting the following criteria were included: (1) randomized controlled trials (RCTs) investigating myopia prevention; (2) reports of data on myopia incidence, axial length (AL), or spherical equivalent (SE) measurements; and (3) subjects were children without myopia as defined by cycloplegic SE ≥ −0.50, undergoing ≥ 1 of the following interventions: atropine, red light therapy, specific exercise regimens, outdoor activities, specialized spectacle lenses, or participation in myopia awareness programs, over an intervention period ≥6 months.

Studies were excluded if they: (1) involved only children with existing myopia; (2) included participants with congenital or severe ophthalmological conditions or a history of eye surgery, which could affect outcomes; or (3) included overlapping participant populations, such as studies using the same cohort or a dataset reported in multiple publications.

### 2.3. Outcome Measures

The primary outcomes were mean annual changes in refraction (diopters/year) and AL (mm/year). For comparisons, values represent the differences in outcomes between intervention and placebo groups. In terms of refractive error, a positive mean difference (MD) indicates less myopia progression (favorable effect), while in terms of AL, a negative MD indicates less axial elongation (favorable effect).

Secondary outcomes included the risk ratio (RR) of annual myopia incidence. We also extracted data on adverse effects, such as photophobia or allergic conjunctivitis, where reported.

### 2.4. Data Sources and Literature Searches

A comprehensive search strategy with no language restrictions across multiple databases, including PubMed, Embase, and Cochrane CENTRAL, was performed independently by two authors (Ssu-Hsien Lee and Bor-Yuan Tseng) from the inception of the databases to November 2024. MeSH terms and relevant EMBASE search terms were used when available. In addition, the reference lists from relevant reviews were manually screened to ensure inclusivity. The detailed search strategy is available in Supplementary Table S1.

### 2.5. Risk-of-Bias Assessment

The methodological quality of the included RCTs was evaluated using the Cochrane risk-of-bias tool for randomized trials, version 2 (RoB 2.0) [18]. The tool assesses five main items: the randomization process, adherence to interventions, missing outcome data, outcome measurement, and selective reporting, leading to an overall risk of bias assessment. Two authors (Ssu-Hsien Lee and Bor-Yuan Tseng) performed the assessment independently, with any disagreements resolved through discussion with two additional authors.

### 2.6. Data Extraction

Two authors (Ssu-Hsien Lee and Bor-Yuan Tseng) independently extracted data from the selected studies, including author information, publication year, study design and location, population characteristics, intervention details, outcomes, and adverse events. When essential data were unavailable in the published articles, we contacted the corresponding authors to obtain the original data.

### 2.7. Data Synthesis and Analysis

The NMA was conducted using the netmeta package in R (version 4.4.1), while risk-of-bias plots were generated using the robvis package. A random-effects model under the frequentist framework was used to perform both direct and indirect comparisons among interventions, incorporating both paired and multi-arm trials. To visualize treatment connections and allow for visual comparison, network and forest plots were generated, respectively. Relative treatment effects were calculated using direct and indirect evidence, with effect sizes expressed as risk ratios or mean differences and 95% confidence intervals (CIs). Statistical significance was set at *p* < 0.05. Treatment rankings for each outcome were determined through 10,000 simulations using the P-score method, which assigns scores ranging from 1 (highest) to 0 (lowest). Additionally, to visualize the comparative effectiveness of treatments, a cumulative probability ranking plot was generated.

To test the robustness of the findings, sensitivity analyses were conducted by excluding studies with a high risk of bias, followed by another NMA on the remaining studies. Inconsistency was assessed globally by comparing direct and indirect evidence with a threshold of *p* > 0.05, indicating no evidence of inconsistency [19]. Local inconsistency was evaluated through node-splitting methods, with *p* > 0.05 indicating no local inconsistency [20]. Heterogeneity was assessed using the I^2^ statistic, with 25%, 50%, and 75% thresholds indicating low, moderate, and high heterogeneity, respectively. Potential publication bias was examined through comparison-adjusted funnel plots and the Egger’s test, with a *p* < 0.10 suggesting publication bias.

### 2.8. Data and Resource Availability

All data generated or analyzed during this study are included in the published article and its online Supplementary Files.

## 3. Results

### 3.1. Literature Search

**Figure 1:**
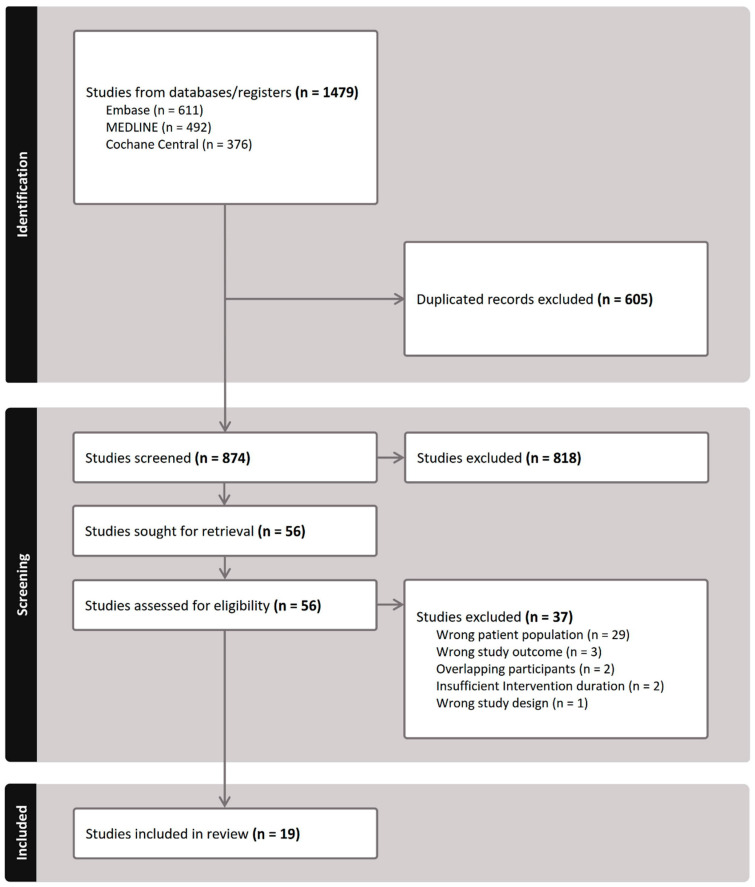
PRISMA flowchart illustrating the study selection for inclusion in the network meta-analysis.

Figure 1 illustrates our literature search and study selection process. Following PRISMA guidelines [17], we initially identified 1479 studies. After removing duplicates and screening titles and abstracts, 56 studies were selected for full-text review. Ultimately, 19 studies met the eligibility criteria and were included in the NMA. The search keywords and exclusion rationale are detailed in Tables S1 and S2, respectively. Additionally, Figures S1–S3 present comparison-adjusted funnel plots and Egger’s test results for SE (*p* = 0.254), AL (*p* = 0.941), and myopia incidence (*p* = 0.004). No publication bias was detected for SE and AL; however, there was evidence of publication bias in myopia incidence.

### 3.2. Characteristics of the Included Studies

**Figure 2:**
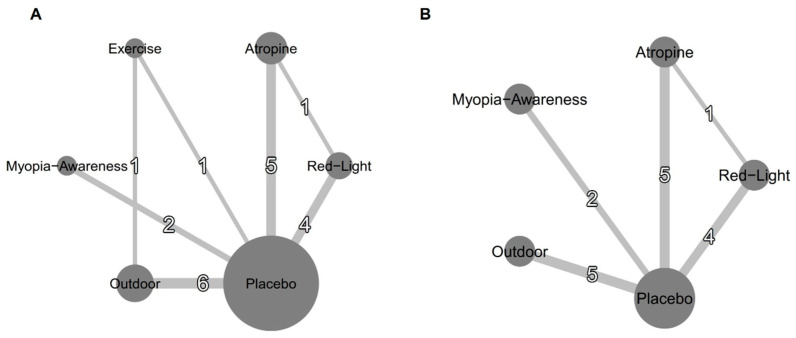
Network plots for efficacy: (**A**) mean annual refraction change and (**B**) mean annual axial length change. Treatments with direct comparisons are connected by lines, with line thickness proportional to the number of trials evaluating each comparison.

Table 1 summarizes the characteristics of the included studies; Figure 2 presents the network plot for SE and AL interventions, and Figure S4 shows the network plots for myopia incidence. The analysis included 19 studies assessing interventions such as low-level red light, low-dose atropine, outdoor activities, myopia awareness programs, and exercise. The studies collectively involved 14,173 children without myopia, with an average age of 6.77 ± 0.67 years, and 52.63% male participants. The baseline cycloplegic SE and AL were 0.88 ± 0.90 and 22.25 ± 0.73, respectively. All studies were conducted in Asian countries, including India, China, and Taiwan, with interventions lasting 6–36 months.

### 3.3. Results of Risk-of-Bias Assessment

Figures S5 and S6 present individual and summary results of RoB 2.0 assessments, respectively. Some studies lacked details on the randomization process, and interventions such as outdoor activities were generally not blinded. Additionally, some studies lacked pre-registration. However, the use of objective outcome measures helps minimize the influence of subjective biases, thereby reducing the overall potential for bias in the study results. Overall, most assessed studies were at risk for overall bias, while the remaining ones demonstrated a low risk of bias in other domains.

### 3.4. Primary Outcome: SE and AL

**Figure 3:**
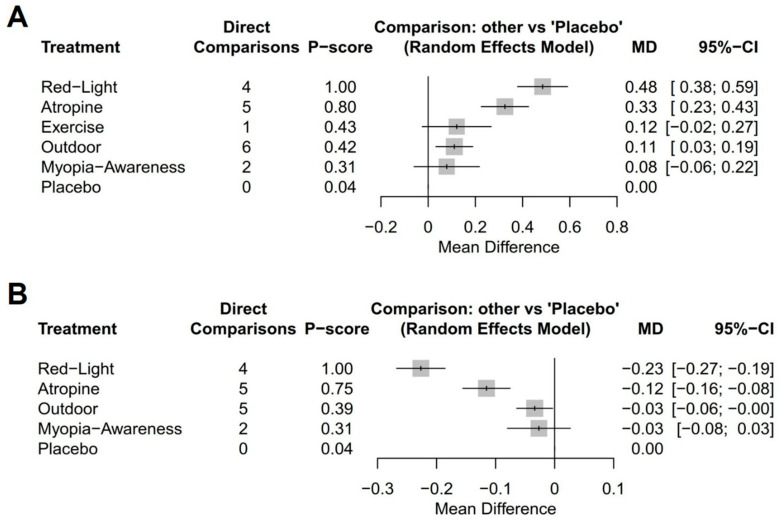
Forest plots of network meta-analysis using placebo as reference intervention: (**A**) mean annual refraction change and (**B**) mean annual axial length change.

Among the interventions evaluating annual refraction changes compared to placebo (Figure 3A), low-level red light demonstrated the most significant improvement (MD, 0.48 D; 95% CI, 0.38–0.59 D), followed by low-dose atropine (MD, 0.33 D; 95% CI, 0.23–0.43 D), outdoor activities (MD, 0.11 D; 95% CI, 0.03–0.19 D), exercise (MD, 0.12 D; 95% CI, −0.02–0.27 D), and a myopia awareness program (MD, 0.08 D; 95% CI, −0.06–0.22 D). For the annual AL change (Figure 3B), low-level red light again showed the greatest benefit (MD, −0.23 mm; 95% CI, −0.27 to −0.19 mm), followed by low-dose atropine (MD, −0.12 mm; 95% CI, −0.16 to −0.08 mm), outdoor activities (MD, −0.03 mm; 95% CI, −0.06–0.00 mm), and a myopia awareness program (MD, −0.03 mm; 95% CI, −0.08–0.03 mm).

**Figure 4:**
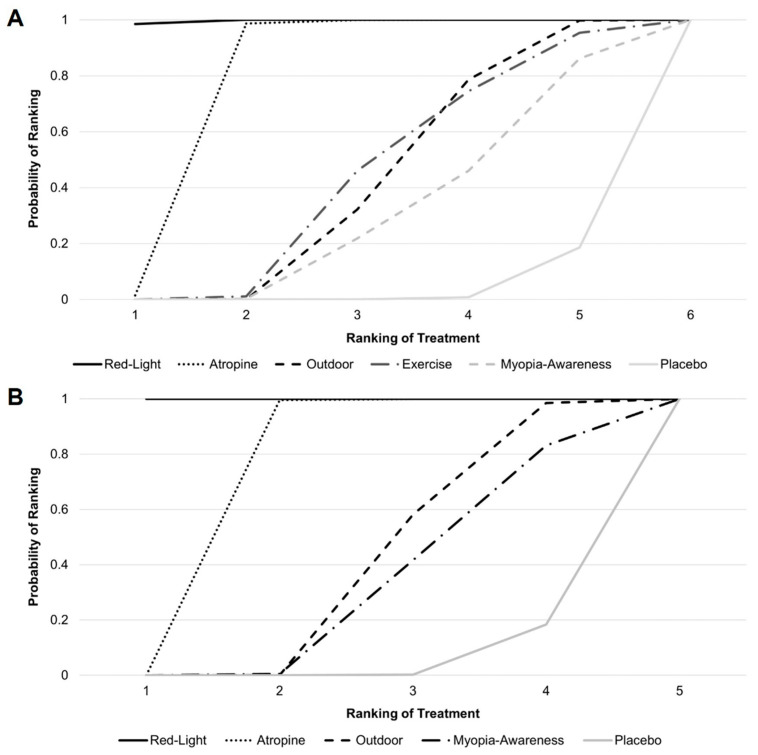
Cumulative probability ranking for (**A**) mean annual refraction change and (**B**) mean annual axial length change.

Figure 4 presents the cumulative probability ranking results based on 10,000 simulations. For mean annual refraction change, low-level red light ranked highest, followed by atropine, outdoor activities, exercise, myopia awareness, and placebo, with rankings determined by the speed at which each intervention reached a cumulative probability of 1. For mean annual axial length change, low-level red light remained the top-ranked intervention, followed by atropine, outdoor activities, myopia awareness, and placebo.

**Figure 5:**
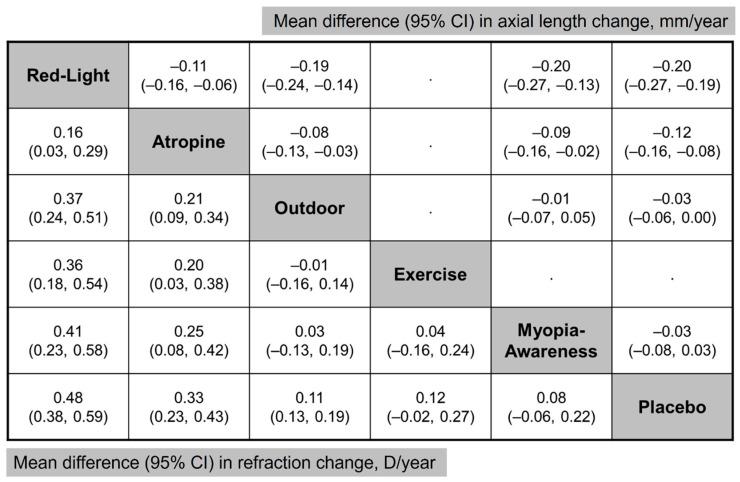
Network meta-analysis comparing all interventions. (**Lower left**) Mean difference in refraction change and (**Upper Right**) mean difference in axial length change. Treatment comparisons are read from left to right.

Figure 5 provides an NMA comparison of all interventions, illustrating the differences in efficacy among treatment methods. For example, atropine demonstrated greater myopia control efficacy than the myopia awareness program, with an MD of 0.25 D (95% CI, 0.08–0.42 D) in annual refraction change and −0.09 mm (95% CI, −0.16 to −0.02 mm) in annual axial length change.

### 3.5. Secondary Outcome: Myopia Incidence and Adverse Events

Figures S7 and S8 show the forest plot and cumulative probability rankings for myopia incidence compared to placebo, respectively. Low-dose atropine was the most effective in reducing myopia incidence (RR, 0.55; 95% CI, 0.41–0.75), followed by low-level red light (RR, 0.59; 95% CI, 0.45–0.79), outdoor activities (RR, 0.82; 95% CI, 0.70–0.97), and a myopia awareness program (RR, 0.88; 95% CI, 0.71–1.09). The results of the comparison among all interventions are summarized in Table S3.

Regarding adverse events, only studies involving low-level red light and low-dose atropine reported safety data, as shown in Tables S4 and S5. In studies using red light therapy, most patients reported no adverse events. However, two patients experienced afterimages lasting >6 min [26], and two others reported intolerance to bright light and initial dry eye symptoms [34]. In atropine studies [10,28,32,33], photophobia occurred more frequently in the low-dose atropine group than in the placebo group, whereas the incidence of allergic conjunctivitis was similar between groups.

### 3.6. Inconsistency Test and Sensitivity Analyses

Figures S9–S11 show the results of inconsistency testing using node-splitting plots for SE, AL, and myopia incidence, respectively. All available comparisons yielded *p*-values > 0.05, indicating no significant inconsistency between direct and indirect comparisons.

We also performed sensitivity analysis after removing two studies with a high risk of bias and subsequently performing an NMA. The results indicated consistent rankings and clinical significance for all interventions, as illustrated in Figures S12–S14 for SE, AL, and myopia incidence, respectively. These results did not deviate from the main findings, confirming the robustness of the analysis.

## 4. Discussion

This NMA is the first comprehensive evaluation of the efficacy and safety of various myopia prevention strategies specifically targeting at-risk children without myopia, addressing a critical research gap. By synthesizing data from 19 studies involving over 14,173 children, our findings provide a robust comparative evaluation of interventions aimed at preventing refractive progression, AL, and myopia onset. Among the evaluated interventions, low-level red light therapy and low-dose atropine demonstrated the greatest efficacy in mitigating refractive and AL changes, both demonstrating significant efficacy in reducing the incidence of myopia. Non-invasive strategies such as outdoor activities and myopia awareness programs showed moderate efficacy. Importantly, none were associated with serious adverse effects, with only minor, self-limiting side effects reported with red light therapy and low-dose atropine. These findings underscore the safety and feasibility of these interventions.

Previous research primarily focused on treating myopic children using atropine and specially designed lenses [8]. In contrast, preventing myopia in non-myopic children involves distinct protective strategies, such as outdoor activities [14]. Notably, outdoor activities have been shown to be more effective in preventing the onset of myopia rather than halting its progression in myopic children [13]. Although the exact mechanisms underlying the protective effects of outdoor activities remain unclear, high ambient light levels may stimulate dopamine release in the retina, regulating eye growth [36]. Alternatively, the diverse visual stimuli and distance-focused environments encountered outdoors may play a role [37]. National initiatives, such as Taiwan’s “Tian-Tian 120” program, which encourages 2 h of daily outdoor activity, highlight the potential of such interventions [38]. However, promoting outdoor activities remains challenging, particularly in urban areas [39], where compliance is often low [25]. As a result, some countries are exploring more feasible alternatives, such as classrooms designed with outdoor views [40].

Children with risk factors such as prolonged screen time, parental myopia, and limited outdoor exposure are particularly susceptible to developing myopia. Early identification of these at-risk children is crucial for implementing preventive strategies that can mitigate refractive progression and axial elongation before myopia fully manifests. A significant proportion of preschool-aged children (5–6 years old) fall into this high-risk category, as demonstrated by a study in Taiwan where 52.0% of children in this age group were in a pre-myopic stage [41]. Additionally, 37.3% of children were hyperopic (SE > 0.75 D); among these, 58.4% had myopic parents, a well-established and strong risk factor for myopia [42,43]. Combined, these findings suggest that nearly 80% of preschool-aged children are either at risk of developing myopia or already exhibit signs of pre-myopia [41]. This growing focus on children at risk of myopia has led to increasing attention on the concept of pre-myopia itself. Recent interventions, such as low-level red light therapy and low-dose atropine, have primarily targeted pre-myopic children. While the definition of pre-myopia remains under debate [10,44], the International Myopia Institute describes it as a refractive state of ≤+0.75 D and >−0.50 D in children with quantifiable risk factors, such as age and parental myopia [45]. While both interventions show promise in managing pre-myopia, concerns persist regarding their potential to disrupt emmetropization in hyperopic children [46]. Determining the optimal timing for initiating these therapies in pre-myopic and hyperopic children remains a critical area for future research. Addressing these concerns will help refine intervention strategies, ensuring both efficacy and safety in myopia prevention.

Compliance is a critical determinant of success in myopia prevention strategies [47]. For example, red light therapy benefits from internet-connected devices that enable adherence monitoring [21], with studies reporting compliance rates of approximately 80% [21,48]. However, logistical barriers, such as the need for clinic visits or device rentals, can hinder widespread adoption [26]. Similarly, while low-dose atropine is generally well-tolerated, with subclinical and reversible side effects [49], adherence may vary due to parental concerns, potentially affecting its consistent use [47,50]. Notably, all included atropine studies, except for LAMP studies [10], utilized 0.01% atropine. While higher atropine concentrations are associated with greater efficacy, they also carry an increased risk of adverse effects. Determining the optimal dosage requires further investigation to balance effectiveness and safety. Other interventions, such as outdoor activities, require significant lifestyle modifications. Technology-based solutions, such as smartwatches and mobile applications [51], could improve adherence by tracking activity levels, promoting outdoor exposure, and encouraging healthy habits. Integrating awareness programs could further enhance compliance and maximize the benefits of such strategies [29]. Ultimately, a shared decision-making approach involving parents, children, and healthcare providers is essential to tailor interventions to individual preferences and lifestyles [52].

Rebound effects pose a significant challenge in long-term myopia prevention. While both red light therapy and low-dose atropine effectively reduce refractive progression, axial elongation, and myopia incidence, both are associated with rebound effects upon cessation [53,54]. Despite this limitation, their overall efficacy remains superior to no intervention [49,54]. Currently, there is a lack of standardized cessation guidelines, so tapering or timing parameters remain unclear. Despite the limited evidence in this area, it appears that even non-pharmacological approaches, such as outdoor activities, exhibit rebound effects [55]. Addressing these gaps requires further research to develop comprehensive, long-term strategies for mitigating rebound effects across all intervention modalities.

Emerging technologies, including artificial intelligence (AI), offer promising opportunities to improve myopia prevention [56]. AI tools could facilitate the early identification of high-risk individuals and support personalized intervention strategies, such as red light therapy or low-dose atropine. Wearable devices and mobile applications could further bolster adherence by monitoring outdoor exposure, reducing near work, and promoting healthy behaviors. Coupled with national and global investment in prevention strategies, these innovations could significantly reduce the global burden of myopia.

### Limitations

While our study provides valuable insights, several limitations should be acknowledged. First, the variability in study quality, particularly the inconsistent reporting of randomization and blinding—especially for interventions like outdoor activities—could be a potential bias. Nevertheless, the objective nature of the measured outcomes may mitigate some of this bias. Second, evidence of publication bias for myopia incidence was identified, necessitating cautious interpretation. Third, the heterogeneity of study populations, including children with varying refractive statuses such as pre-myopic and hyperopic individuals, may limit the precision of subgroup analyses. Further research is needed to better define at-risk children and refine preventive strategies accordingly. However, this diversity also reflects the broader target population subject to preventive strategies. Additionally, the lack of long-term data on rebound effects after treatment cessation and inconsistent reporting of compliance data constrain our understanding of the real-world applicability of these interventions. Another notable limitation is that the included studies were mostly conducted in Asian populations and, thus, subject to cultural and environmental factors limiting the generalizability of the findings. Further research in other regions is needed. Finally, while this study evaluated the efficacy of individual interventions, the potential synergistic effects of various strategies could not be assessed due to insufficient data. Future research should explore integrated approaches to optimize preventive outcomes. Expanding clinical trials to include more diverse populations and establishing standardized protocols for early intervention, treatment cessation, and rebound effect assessment are crucial to addressing these gaps.

## 5. Conclusions

According to our results, low-level red light therapy and low-dose atropine are the most effective interventions for preventing myopia in children without pre-existing myopia, with outdoor activities and myopia awareness programs offering moderate benefits. All interventions showed favorable safety profiles. These findings provide robust evidence to guide clinical decision-making and emphasize the importance of balancing efficacy, safety, and compliance in implementing myopia prevention strategies.

## Figures and Tables

**Table 1:** Summary of included study characteristics.

Study	Follow-Up (Months)	Intervention	Sample Size	Male (%)	Age (Years)	Baseline SE (D)	Baseline AL (mm)	Region
Cao 2024 [21]	12	Red Light (3 min/BID)	56	46.4	9.1 ± 2.0	0.2 ± 0.6	23.1 ± 0.8	China
		Placebo	56	48.2	9.0 ± 1.9	0.3 ± 0.5	23.1 ± 0.7	
D. Wang 2023 [22]	9	Outdoor (600 min/week)	702	50.4	9.2 ± 0.6	0.6 ± 1.1	23.1 ± 0.8	China
		Placebo	728	47.3		0.6 ± 1.1	23.1 ± 0.8	
G. Liu 2024 [23]	12	Red Light (3 min/BID)	40	55.0	9.0 ± 0.9	0.4 ± 0.3	23.4 ± 0.6	China
		Placebo	36	47.2	8.9 ± 1.1	0.4 ± 0.3	23.3 ± 0.8	
He 2015 [24]	36	Outdoor (200 min/week)	853	52.6	6.6 ± 0.3	1.3 ± 1.0	22.6 ± 0.7	China
		Placebo	726	54.7	6.6 ± 0.3	1.3 ± 0.8	22.7 ± 0.7	
He 2022 [25]	24	Outdoor (200–400 min/week)	3459	53.0	7.3 ± 0.7	1.0 ± 1.0	22.9 ± 0.8	China
		Placebo	1608	53.9	7.2 ± 0.7	1.0 ± 1.0	22.9 ± 0.8	
He 2023 [26]	12	Red Light (3 min/BID)	120	51.1	8.3 ± 1.1	0.1 ± 0.3	23.4 ± 0.7	China
		Placebo	111	48.9	8.3 ± 1.1	0.2 ± 0.3	23.3 ± 0.7	
Hua 2015 [27]	12	Outdoor	87	51.7	10.7 ± 2.4	0.6 ± 0.5	23.1 ± 0.6	China
		Placebo	69	46.4	10.5 ± 2.3	0.6 ± 0.5	23.1 ± 0.7	
Jethani 2022 [28]	24	Atropine (0.01%QD)	30	N/A	7.7 ± 2.1	N/A	20.8 ± 0.6	India
		Placebo	30		7.2 ± 1.9		21.0 ± 0.5	
Li 2021 [29]	24	Myopia Awareness Program	544	54.6	6.3 ± 0.5	1.1 ± 1.0	22.7 ± 0.7	China
		Placebo	700	54.9	6.3 ± 0.5	1.0 ± 1.0	22.7 ± 0.7	
Li 2022 [15]	12	Myopia Awareness Program	114	52.6	8.4 ± 0.3	0.7 ± 1.1	23.1 ± 0.7	China
		Placebo	110	57.1	8.4 ± 0.3	0.4 ± 1.3	23.2 ± 0.8	
Liao 2023 [16]	12	Outdoor (180 min/week)	25	52.0	6.4 ± 0.5	1.5 ± 0.2	N/A	China
		Exercise (180 min/week)	24	58.3	6.5 ± 0.5	1.6 ± 0.2		
		Placebo	25	52.0	6.5 ± 0.5	1.5 ± 0.2		
Shang 2024 [30]	6	Red Light (3 min/BID)	32	46.9	8.8 ± 1.2	−0.2 ± 0.3	23.6 ± 0.8	China
		Atropine (0.01%QD)	30	53.3	8.8 ± 1.1	−0.2 ± 0.3	23.6 ± 0.7	
Tong 2024 [31]	36	Myopia Awareness Program	163	51.0	9.4 ± 1.5	N/A	N/A	China
		Placebo	159	52.5	9.2 ± 1.2			
W. Wang 2023 [32]	6	Atropine (0.01%QD)	26	46.7	8.6 ± 1.7	−0.2 ± 0.3	23.6 ± 0.8	China
		Placebo	25	50.0	8.5 ± 1.7	−0.2 ± 0.3	23.6 ± 0.8	
Wu 2018 [14]	12	Outdoor (660 min/week)	235	55.1	6.3 ± 0.5	0.4 ± 1.1	22.8 ± 0.8	Taiwan
		Placebo	385	50.3		0.3 ± 1.0	22.8 ± 0.8	
Yam 2023 [10]	24	Atropine (0.01%QD)	122	49.1	6.9 ± 1.4	0.5 ± 0.3	22.9 ± 0.7	Hong Kong
		Atropine (0.05%QD)	116	50.6	6.9 ± 1.4	0.5 ± 0.3	22.8 ± 0.7	
		Placebo	115	50.3	6.8 ± 1.3	0.5 ± 0.3	22.8 ± 0.6	
Yu 2022 [33]	6	Atropine (0.01%QD)	26	42.3	8.4 ± 1.5	−0.2 ± 0.3	23.5 ± 0.7	China
		Placebo	25	48.0	8.7 ± 1.9	−0.2 ± 0.3	23.6 ± 0.8	
Z. Liu 2024 [34]	12	Red Light (3 min/BID)	43	55.8	9.0 ± 1.3	0.2 ± 0.4	23.6 ± 0.8	China
		Placebo	42	52.4	9.0 ± 1.5	0.3 ± 0.4	23.3 ± 0.7	
Zhang 2024 [35]	36	Myopia Awareness Program	1138	53.9	6.9 ± 0.7	1.0 ± 0.9	22.9 ± 0.7	China
		Placebo	1238	55.1	6.8 ± 0.7	1.0 ± 0.9	22.9 ± 0.7	

Note: min: Minute; N/A: Not available; QD: Once a day; BID: Twice a day; All data shown are means ± SDs, if not marked otherwise.

## Data Availability

Data are contained within the article or Supplementary Materials. Further inquiries can be directed to the corresponding author.

## Supplementary Materials

The following supporting information can be downloaded at https://www.mdpi.com/article/10.3390/jcm14051665/s1, Table S1. Keywords and search results across different databases, Table S2. Excluded studies and corresponding reasons for exclusion, Table S3. Network meta-analysis comparing interventions for myopia, with the risk ratio of myopia incidence, Table S4. Detailed adverse events reported in studies of low-level red light therapy, Table S5. Detailed adverse events reported in studies of low-dose atropine therapy, Figure S1. Funnel plot for SE, Figure S2. Funnel plot for AL, Figure S3. Funnel plot for myopia incidence, Figure S4. Network plot for myopia incidence, Figure S5. Detailed quality assessment of included studies using the Cochrane risk of bias 2 (RoB 2.0) tool, Figure S6. Summary results from the Cochrane risk of bias 2 (RoB 2.0) tool, Figure S7. Forest plots of the network meta-analysis with placebo as the referent intervention for myopia incidence, Figure S8. Cumulative probability ranking results for myopia incidence, Figure S9. Node-splitting plot for SE, Figure S10. Node-splitting plot for AL, Figure S11. Node-splitting plot for myopia incidence, Figure S12. Sensitivity analysis for SE, Figure S13. Sensitivity analysis for AL, Figure S14. Sensitivity analysis for myopia incidence.
