# Supplementary material for: Efficacy of Myopia Prevention in At-Risk Children: A Systematic Review and Network Meta-Analysis
Source: J Clin Med. 2025 Feb 28;14(5):1665. doi: 10.3390/jcm14051665 (PMC11900620; doi:10.3390/jcm14051665)
Supplement: Supplementary file 1 [file jcm-14-01665-s001.zip › jcm-3464766-supplementary.pdf]

Supplementary Material

Efficacy of Myopia Prevention Methods in At-Risk Children: A Systematic Review and Network Meta-Analysis

Ssu-Hsien Lee,[1] Bor-Yuan Tseng,[1] Jen-Hung Wang,[2] Cheng-Jen Chiu [3,4*]

[1] School of Medicine, Tzu Chi University, Hualien, Taiwan.

[2] Department of Medical Research, Buddhist Tzu Chi General Hospital, Hualien, Taiwan

[3] Department of Ophthalmology and Visual Science, Tzu Chi University, Hualien, Taiwan.

[4] Department of Ophthalmology, Hualien Tzu Chi Hospital, the Buddhist Tzu Chi Medical Foundation, Hualien, Taiwan.

*Correspondence: Email: drcjchiu@outlook.com.tw; Phone:(886)-3-8565526; Fax: (886)-3-8577161; Address: No. 707, Section 3, Zhongyang Rd, Hualien City, Hualien County, 970

Table of Contents

Table S1. Keywords and search results across different databases. 3

Table S2. Excluded studies and corresponding reasons for exclusion. 5

Table S3. Network meta-analysis comparing interventions for myopia, with the risk ratio of myopia incidence. 8

Table S4. Detailed adverse events reported in studies of low-level red-light therapy 8

Table S5. Detailed adverse events reported in studies of low-dose atropine therapy 9

Figure S1. Funnel plot for SE 10

Figure S2. Funnel plot for AL. 10

Figure S3. Funnel plot for myopia incidence. 11

Figure S4. Network plot for myopia incidence. 12

Figure S5. Detailed quality assessment of included studies using the Cochrane risk of bias 2 (RoB 2.0) tool. 13

Figure S6. Summary results from the Cochrane risk of bias 2 (RoB 2.0) tool. 14

Figure S7. Forest plots of the network meta-analysis with placebo as the referent intervention for myopia incidence. 15

Figure S8. Cumulative probability ranking results for myopia incidence. 15

Figure S9. Node-splitting plot for SE. 16

Figure S10. Node-splitting plot for AL. 17

Figure S11. Node-splitting plot for myopia incidence. 18

Figure S12. Sensitivity analysis for SE. 19

Figure S13. Sensitivity analysis for AL. 19

Figure S14. Sensitivity analysis for myopia incidence. 19

References 20

Table S1. Keywords and search results across different databases.

Database (Results)	#	Search Terms
PubMed (492)	#1	"Child"[MeSH] OR child* OR pediatric* OR paediatric*
	#2	"Myopia/prevention and control"[MeSH] OR "Myopia Incidence" OR "Myopia Onset" OR "Myopia Control" OR "Myopia Prevention" OR "Preventing Myopia" OR "Prevention of Myopia" OR premyopia OR premyopic OR emmetropia OR emmetropic OR "Non-Myopic" OR "Non-Myopia"
	#3	"Atropine"[MeSH] OR atropine OR "Red Light" OR "Phototherapy"[MeSH] OR phototherapy OR "Education"[MeSH] OR education OR "Lifestyle Intervention*" OR "Optical Intervention*" OR "Outdoor Activity" OR "Time Outdoors" OR "Spectacle Lenses" OR "Contact Lenses" OR orthokeratology OR "Myopia Control" OR "Myopia Prevention"
	#4	effectiveness OR efficacy OR safety OR "adverse effect*" OR "Treatment Outcome"[MeSH] OR "Disease Progression"[MeSH] OR "Risk Factors"[MeSH] OR outcome* OR incidence OR progression OR onset OR "Myopia Progression" OR "Axial Length" OR "Spherical Equivalent" OR refraction
	#5	(randomized controlled trial[pt] OR controlled clinical trial[pt] OR randomized[tiab] OR placebo[tiab] OR "Drug Therapy"[Subheading] OR randomly[tiab] OR trial[tiab] OR groups[tiab]) NOT (animals[mh] NOT humans[mh])
	#6	#1 AND #2 AND #3 AND #4 AND #5
Embase (611)	#1	'child'/exp OR child* OR pediatric* OR paediatric*
	#2	'myopia prevention'/exp OR 'myopia incidence' OR 'myopia onset' OR 'myopia control' OR 'preventing myopia' OR 'prevention of myopia' OR premyopia OR premyopic OR emmetropia OR emmetropic OR 'non-myopic' OR 'non-myopia'
	#3	'atropine'/exp OR atropine OR 'red light' OR 'phototherapy'/exp OR phototherapy OR 'light therapy'/exp OR 'education'/exp OR education OR 'lifestyle intervention*' OR 'optical intervention*' OR 'outdoor activity' OR 'time outdoors' OR 'spectacle lenses' OR 'contact lenses' OR orthokeratology OR 'myopia control' OR 'myopia prevention

	#4	effectiveness OR efficacy OR safety OR 'adverse effect*' OR 'treatment outcome'/exp OR 'disease progression'/exp OR 'risk factor'/exp OR outcome* OR incidence OR progression OR onset OR 'myopia progression' OR 'axial length' OR 'spherical equivalent' OR refraction
	#5	('randomized controlled trial'/exp OR 'controlled clinical trial'/exp OR random*:ti,ab OR placebo:ti,ab OR 'drug therapy'/exp OR randomly:ti,ab OR trial:ti,ab OR groups:ti,ab) NOT (('animal'/exp OR 'animal') NOT ('human'/exp OR 'human'))
	#6	#1 AND #2 AND #3 AND #4 AND #5
**Cochrane CENTRAL (376)**	#1	child* OR pediatric* OR paediatric*
	#2	"Myopia/prevention and control" OR "Myopia Incidence" OR "Myopia Onset" OR "Myopia Control" OR "Myopia Prevention" OR "Preventing Myopia" OR "Prevention of Myopia" OR premyopia OR premyopic OR emmetropia OR emmetropic OR Non-Myopic OR Non-Myopia
	#3	atropine OR phototherapy OR education OR "Red Light" OR "Light Therapy" OR "Lifestyle Intervention" OR "Optical Intervention" OR "Outdoor Activity" OR "Time Outdoors" OR "Spectacle Lenses" OR "Contact Lenses" OR orthokeratology OR "Myopia Control" OR "Myopia Prevention"
	#4	effectiveness OR efficacy OR safety OR "adverse effect*" OR outcome* OR incidence OR progression OR onset OR "Myopia Progression" OR "Axial Length" OR "Spherical Equivalent" OR refraction
	#5	#1 AND #2 AND #3 AND #4
	#6	Limit to "Trials" within the Cochrane Library.

Table S2. Excluded studies and corresponding reasons for exclusion.

Study	Reason for exclusion
Stable myopia control during 3-year wear of orthokeratology lenses in Danish children [1]	Wrong patient population
One-year efficacy of myopia control by the defocus distributed multipoint lens a multicentric randomised controlled trial [2]	Wrong patient population
The effect of low-concentration atropine combined with auricular acupoint stimulation in myopia control [3]	Wrong patient population
Soft Contact Lenses with Positive Spherical Aberration for Myopia Control [4]	Wrong patient population
Discontinuation of orthokeratology on eyeball elongation (DOEE) [5]	Wrong patient population
Myopia Control Efficacy and Long-Term Safety of a Novel Orthokeratology Lens (MESOK Study)-A Randomized Controlled Clinical Trial Combining Clinical and Tear Proteomics Data [6]	Wrong patient population
Influence of wearing time on myopia control efficacy of spectacle lenses with aspherical lenslets [7]	Wrong patient population
The Hong Kong Progressive Lens Myopia Control Study: Study Design and Main Findings [8]	Wrong patient population
Seasonal variation in myopia progression and axial elongation an evaluation of Japanese children participating in a myopia control trial [9]	Wrong patient population
Myopia Control with Low-Dose Atropine in European Children Six-Month Results from a Randomized, Double-Masked, Placebo-Controlled, Multicenter Study [10]	Wrong patient population
Effects of short-term use of atropine with different concentrations and frequencies on eye safety in children [11]	Insufficient intervention duration
Assessment of myopic rebound effect after discontinuation of treatment with 0.01% atropine eye drops in Japanese school-age children [12]	Wrong patient population
A Randomized Controlled Trial of the Effect of 0.01_ Atropine Eye Drops Combined with Auricular Acupoint Stimulation on Myopia Progression [13]	Wrong patient population
Defocus Incorporated Soft Contact (DISC) lens [14]	Wrong patient population

Long-term myopia control effect and safety in children wearing DIMS spectacle lenses for 6 years [15]	Wrong patient population
Control of myopia using diffusion optics spectacle lenses 4-year results of a multicentre randomised controlled, efficacy and safety study (CYPRESS) [16]	Wrong patient population
Low-concentration atropine eyedrops for myopia control in a multi-racial cohort of Australian children A randomised clinical trial [17]	Wrong patient population
Soft peripheral contact lens for eye elongation control (SPACE) 1-year results of a double-blinded randomized controlled trial [18]	Wrong patient population
Myopia Control Efficacy of Spectacle Lenses With Aspherical Lenslets Results of a 3-Year Follow-Up Study [19]	Wrong patient population
Effect of orthokeratology combined with repeated low-level red-light therapy on progressive myopia in adolescents [20]	Wrong patient population
Superdiluted atropine at 0.01% reduces progression in children and adolescents. A 5 year study of safety and effectiveness [21]	Wrong patient population
Increased outdoor time reduces incident myopia - The Guangzhou outdoor activity longitudinal study [22]	Overlapping participants
Five-year results of atropine 0.01% efficacy in the myopia control in a European population [23]	Wrong patient population
A cluster randomised controlled trial evaluating an incentive-based outdoor physical activity programme to increase outdoor time and prevent myopia in children [24]	Wrong study outcome
Biometric treatment efficacy of 0.01% atropine eye drops analysis of a randomized controlled trial [25]	Wrong patient population
Center-for-Near Extended-Depth-of-Focus Soft Contact Lens for Myopia Control in Children 1-Year Results of a Randomized Controlled Trial [26]	Wrong patient population
One-year results for myopia control of orthokeratology with different back optic zone diameters a randomized trial using a novel multispectral-based topographer [27]	Wrong patient population
A randomized trial of the effects of rigid contact lenses on myopia progression [28]	Wrong patient population
Longitudinal Changes in Choroidal Structure Following Repeated Low-Level Red-Light Therapy for Myopia Control Secondary Analysis of a Randomized Controlled Trial [29]	Wrong patient population

Effects of orthokeratology and spectacle lenses with highly aspherical lenslets on unilateral myopic anisometropia control [30]	Wrong study design
Myopia control effect of Defocus Incorporated Multiple Segments (DIMS) spectacle lens is influenced by baseline relative peripheral refraction [31]	Wrong patient population
Myopia Control Effect Is Influenced by Baseline Relative Peripheral Refraction in Children Wearing Defocus Incorporated Multiple Segments (DIMS) Spectacle Lenses [32]	Wrong patient population
Changes in relative peripheral refraction in children who switched from single-vision lenses to Defocus Incorporated Multiple Segments lenses [33]	Wrong patient population
Six-month repeated irradiation of 650 nm low-level red light reduces the risk of myopia in children a randomized controlled trial [34]	Overlapping participants
Eye growth pattern of myopic children wearing spectacles lenses with aspherical lenslets compared with non-myopic children [35]	Wrong study outcome
Smartwatch Measures of Outdoor Exposure and Myopia in Children [36]	Wrong study outcome
Pseudomyopia treated with auricular point sticking combined with periocular needle-embedding therapy and prevention of true myopia a multicenter randomized controlled trial [37]	Insufficient intervention duration

Table S3. Network meta-analysis comparing interventions for myopia, with the risk ratio of myopia incidence.

Pairwise meta-analysis estimates are presented above the diagonal, while network meta-analysis estimates are provided below the diagonal. Treatment comparisons should be interpreted from left to right.

**Atropine**	-	-	-	0.55 (0.41, 0.75)
0.93 (0.61, 1.42)	**Red-Light**	-	-	0.59 (0.45, 0.79)
0.67 (0.47, 0.95	0.72 (0.52, 1.00)	**Outdoor**	-	0.82 (0.70, 0.97)
0.63 (0.43, 0.91)	0.67 (0.47, 0.96)	0.93 (0.72, 1,22)	**Myopia-Awareness**	0.88 (0.71, 1.09)
0.55 (0.41, 0.75)	0.59 (0.45, 0.79)	0.82 (0.70, 0.97)	0.88 (0.71, 1.09)	**Placebo**

Table S4. Detailed adverse events reported in studies of low-level red-light therapy.

Study	Duration (months)	Red-Light Event Rate	Placebo Event Rate	Details
Cao 2024	12	0/56	0/56	OCT retinal imaging
G.Liu 2024	12	0/40	0/36	Adverse events reported by patients
He 2023	12	2/120	0/111	OCT retinal imaging; two participants reported afterimages lasting >6 minutes
Z.Liu 2024	12	2/43	0/42	Two participants reported intolerance to bright light and dry eye symptoms initially

Table S5. Detailed adverse events reported in studies of low-dose atropine therapy.

Study	Duration (months)	Atropine Event Rate	Placebo Event Rate	Details
Jethani 2022	24	0/30	0/30	Adverse events reported by patients
W.Wang 2023	6	5/26	2/25	Photophobia
Yam 2023 (0.01%)	24	62/261	37/243	52 cases of photophobia and 10 cases of allergic conjunctivitis in the atropine group; 27 cases of photophobia and 10 cases of allergic conjunctivitis in the placebo group
Yam2023 (0.05%)	24	53/252	37/243	43 cases of photophobia and 10 cases of allergic conjunctivitis in the atropine group; 27 cases of photophobia and 10 cases of allergic conjunctivitis in the placebo group
Yu 2022	6	2/43	0/42	Photophobia

Figure S1. Funnel plot for SE.

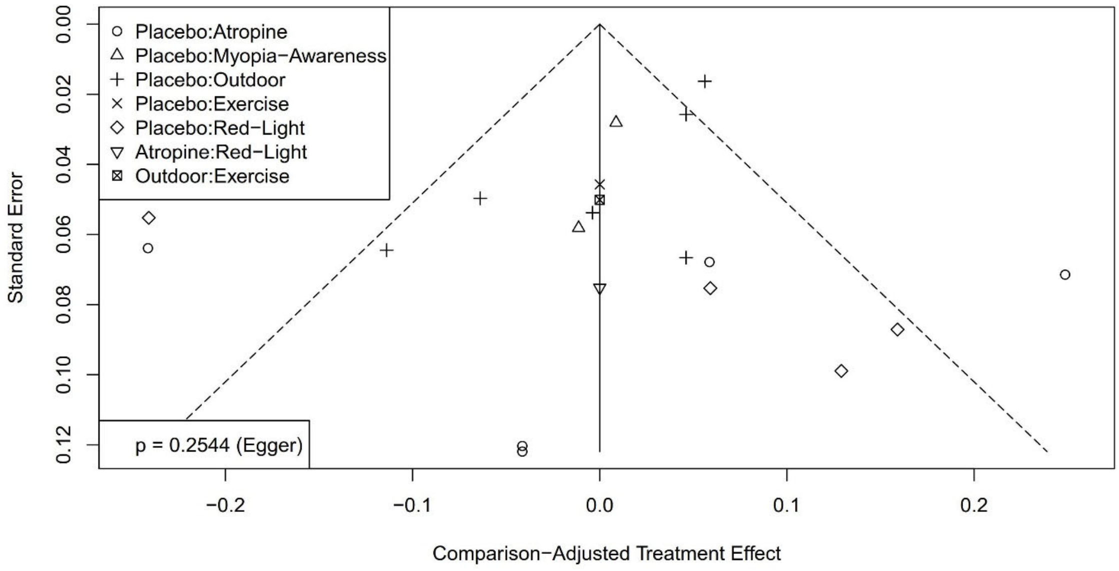

Figure S2. Funnel plot for AL.

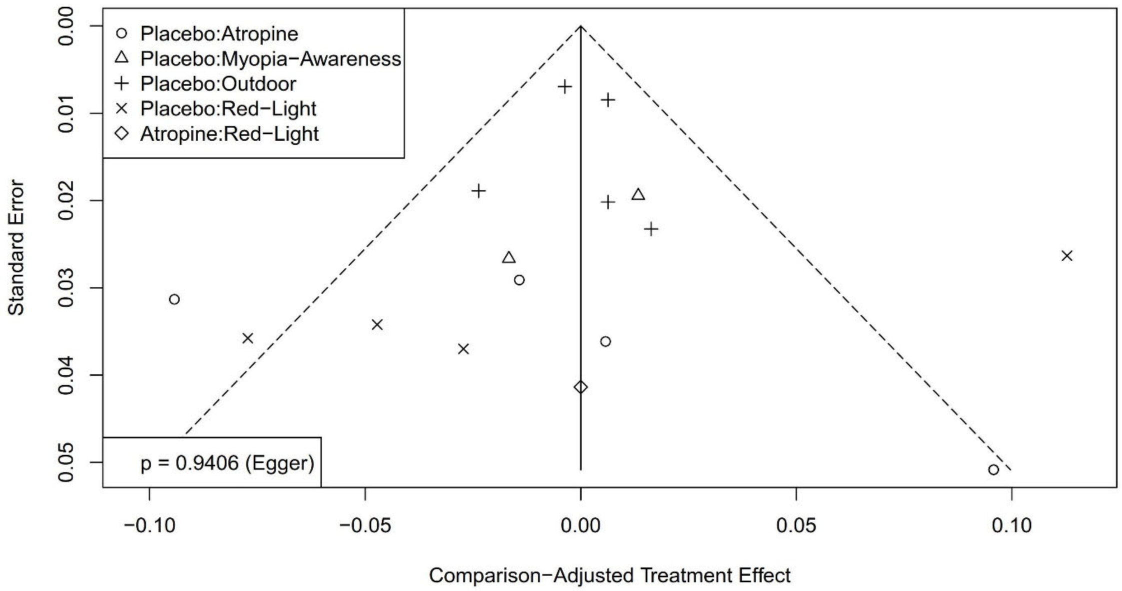

Figure S3. Funnel plot for myopia incidence.

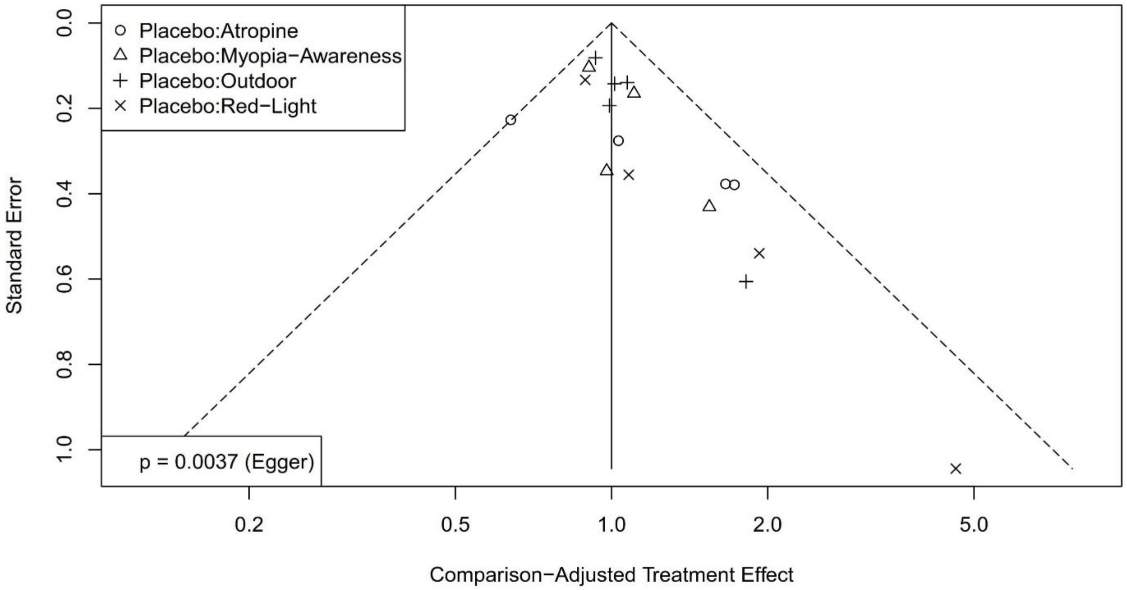

Figure S4. Network plot for myopia incidence.

Treatments with direct comparisons are connected by lines, with line thickness proportional to the number of trials evaluating each comparison.

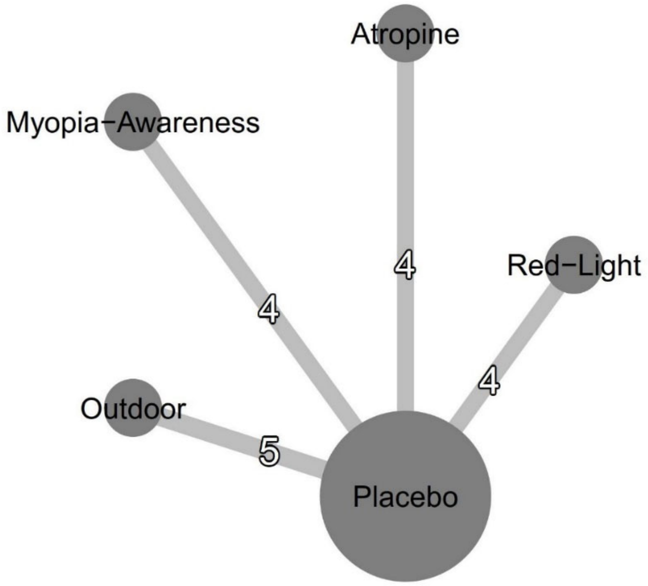

Figure S5. Detailed quality assessment of included studies using the Cochrane risk of bias 2 (RoB 2.0) tool.

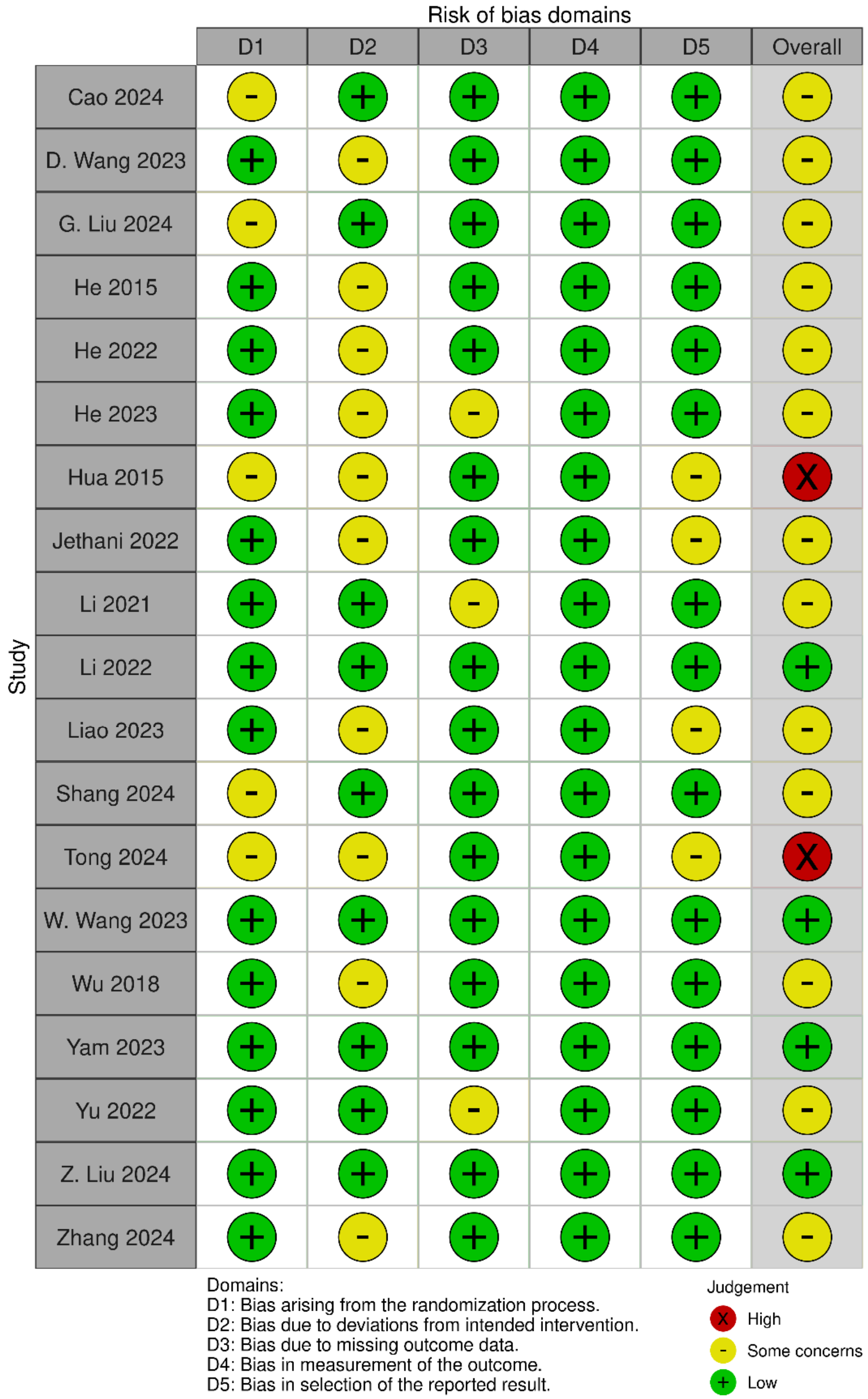

Figure S6. Summary results from the Cochrane risk of bias 2 (RoB 2.0) tool.

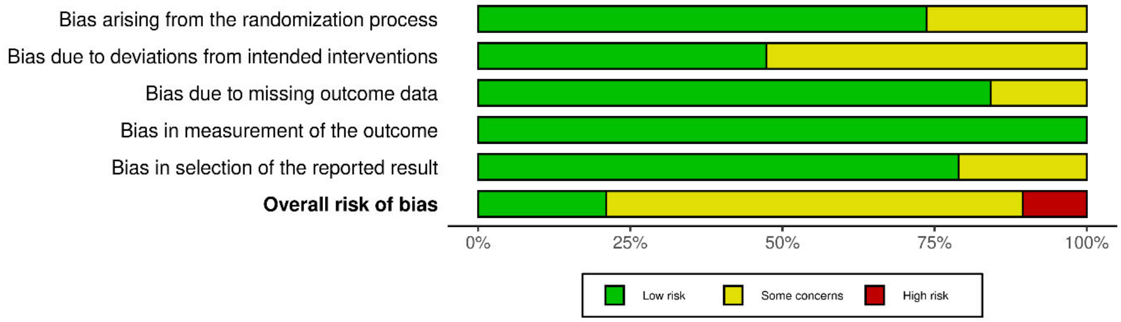

Figure S7. Forest plots of the network meta-analysis with placebo as the referent intervention for myopia incidence.

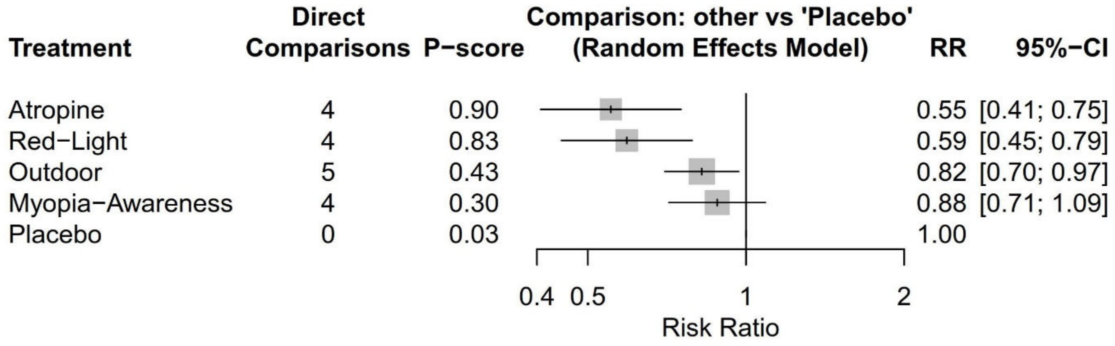

Figure S8. Cumulative probability ranking results for myopia incidence.

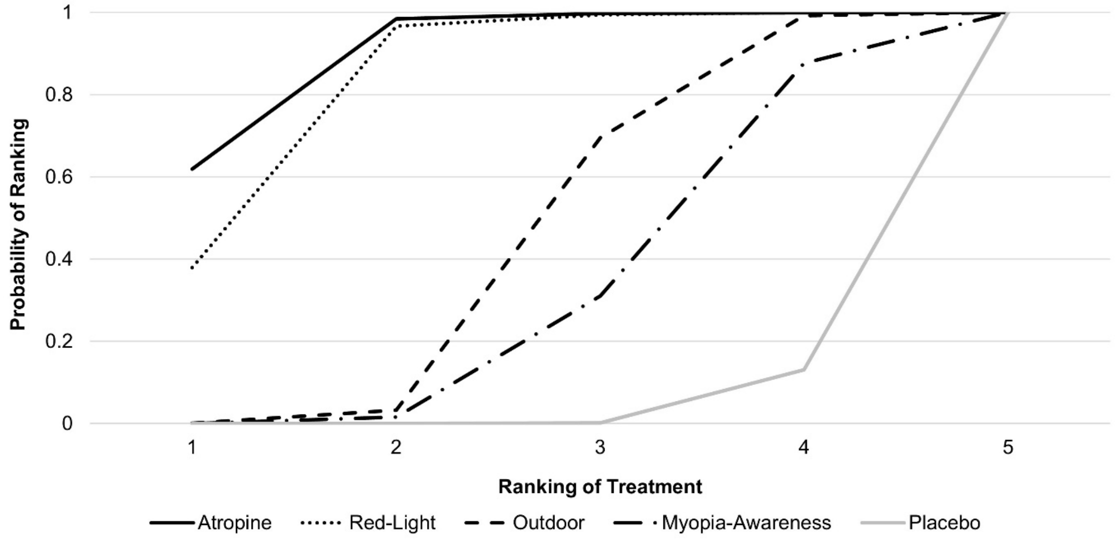

Figure S9. Node-splitting plot for SE.

Comparison	Number of Studies	Direct Evidence	I2	MD	95%-CI
**Atropine:Exercise**					
Direct estimate	0	0			
Indirect estimate				0.20	[0.03; 0.38]
Network estimate				0.20	[0.03; 0.38]
**Atropine:Myopia−Awareness**					
Direct estimate	0	0			
Indirect estimate				0.25	[0.08; 0.42]
Network estimate				0.25	[0.08; 0.42]
**Atropine:Outdoor**					
Direct estimate	0	0			
Indirect estimate				0.21	[0.09; 0.34]
Network estimate				0.21	[0.09; 0.34]
**Atropine:Placebo**					
Direct estimate	5	0.85	85%	0.34	[0.23; 0.45]
Indirect estimate				0.25	[−0.01; 0.51]
Network estimate				0.33	[0.23; 0.43]
**Atropine:Red−Light**					
Direct estimate	1	0.33		−0.22	[−0.45; 0.01]
Indirect estimate				−0.13	[−0.29; 0.03]
Network estimate				−0.16	[−0.29; −0.03]
**Exercise:Myopia−Awareness**					
Direct estimate	0	0			
Indirect estimate				0.04	[−0.16; 0.24]
Network estimate				0.04	[−0.16; 0.24]
**Exercise:Outdoor**					
Direct estimate	1	0.53		−0.02	[−0.22; 0.18]
Indirect estimate				0.04	[−0.17; 0.26]
Network estimate				0.01	[−0.14; 0.16]
**Exercise:Placebo**					
Direct estimate	1	0.55		0.15	[−0.05; 0.35]
Indirect estimate				0.09	[−0.13; 0.30]
Network estimate				0.12	[−0.02; 0.27]
**Exercise:Red−Light**					
Direct estimate	0	0			
Indirect estimate				−0.36	[−0.54; −0.18]
Network estimate				−0.36	[−0.54; −0.18]
**Myopia−Awareness:Outdoor**					
Direct estimate	0	0			
Indirect estimate				−0.03	[−0.19; 0.13]
Network estimate				−0.03	[−0.19; 0.13]
**Myopia−Awareness:Placebo**					
Direct estimate	2	1.00	0%	0.08	[−0.06; 0.22]
Indirect estimate					
Network estimate				0.08	[−0.06; 0.22]
**Myopia−Awareness:Red−Light**					
Direct estimate	0	0			
Indirect estimate				−0.41	[−0.58; −0.23]
Network estimate				−0.41	[−0.58; −0.23]
**Outdoor:Placebo**					
Direct estimate	6	0.92	57%	0.11	[0.03; 0.19]
Indirect estimate				0.17	[−0.11; 0.45]
Network estimate				0.11	[0.03; 0.19]
**Outdoor:Red−Light**					
Direct estimate	0	0			
Indirect estimate				−0.37	[−0.51; −0.24]
Network estimate				−0.37	[−0.51; −0.24]
**Red−Light:Placebo**					
Direct estimate	4	0.83	87%	0.47	[0.35; 0.59]
Indirect estimate				0.56	[0.30; 0.81]
Network estimate				0.48	[0.38; 0.59]

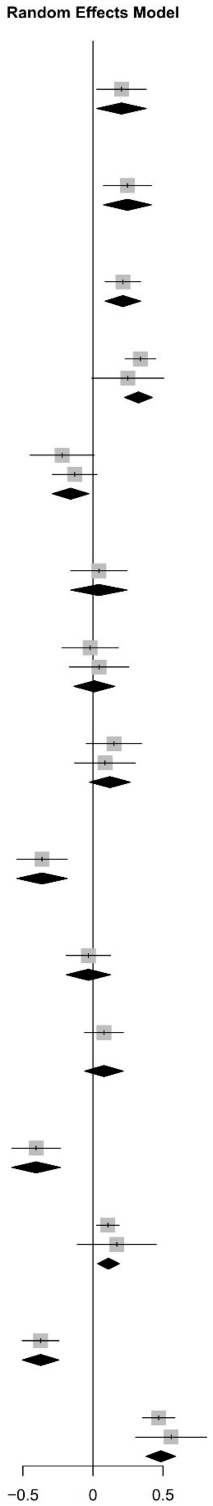

Figure S10. Node-splitting plot for AL.

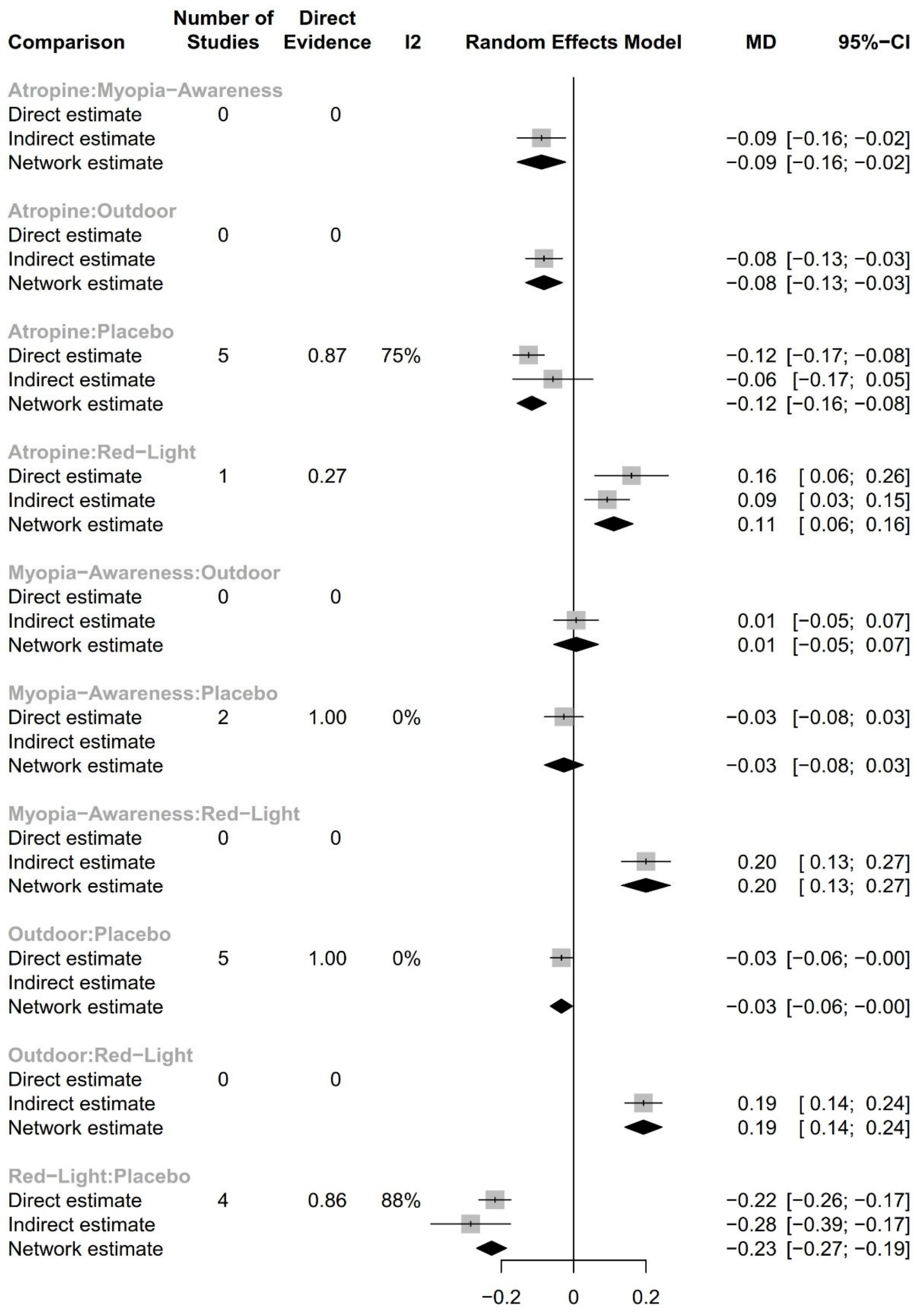

Figure S11. Node-splitting plot for myopia incidence.

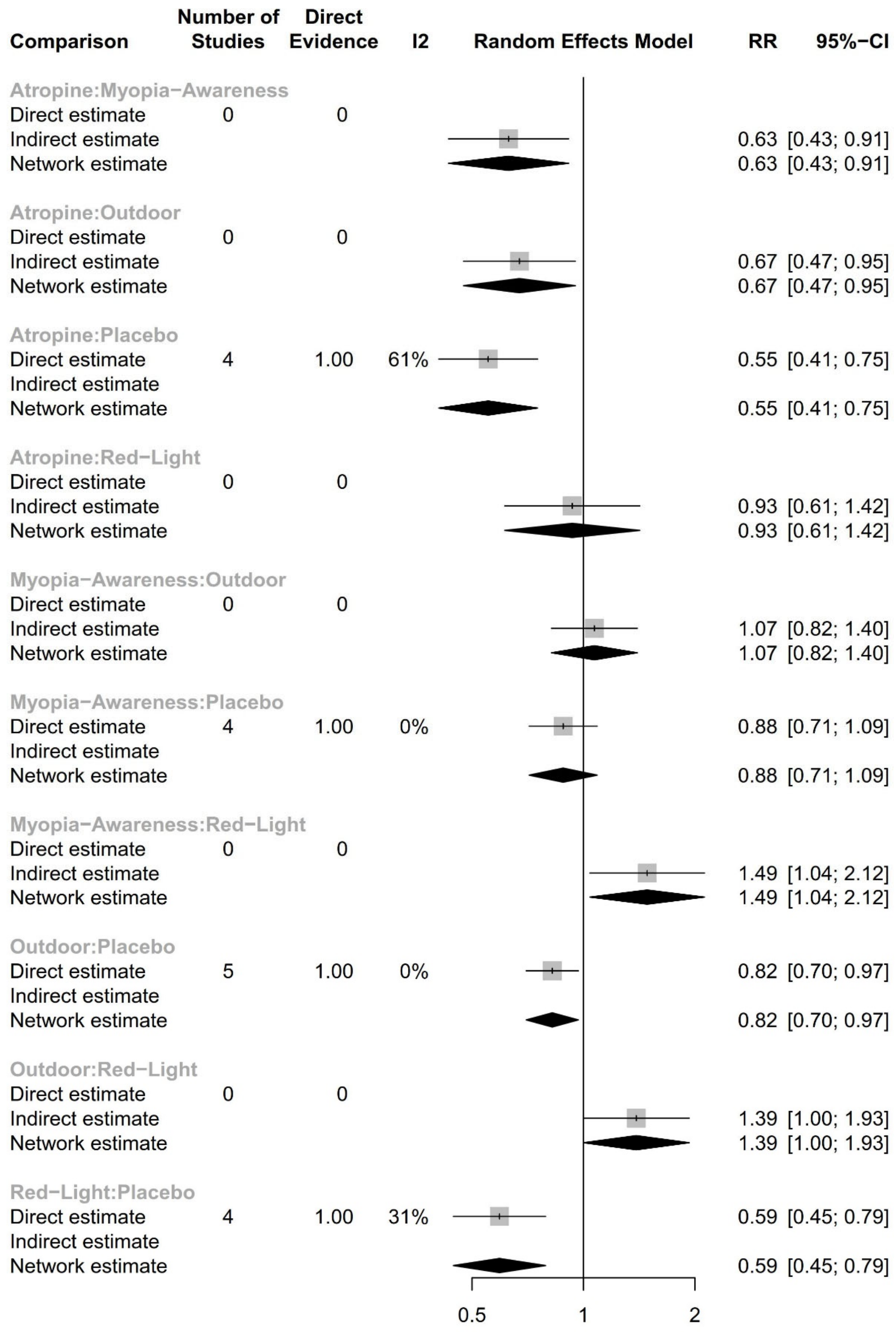

Figure S12. Sensitivity analysis for SE.

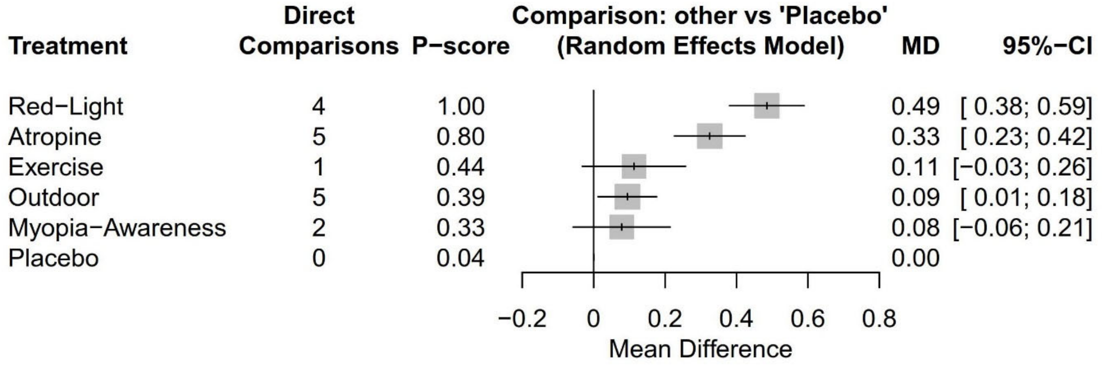

Figure S13. Sensitivity analysis for AL.

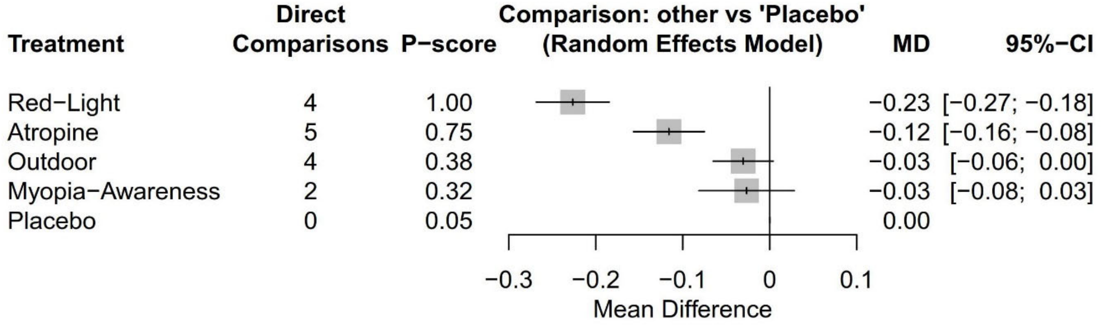

Figure S14. Sensitivity analysis for myopia incidence.

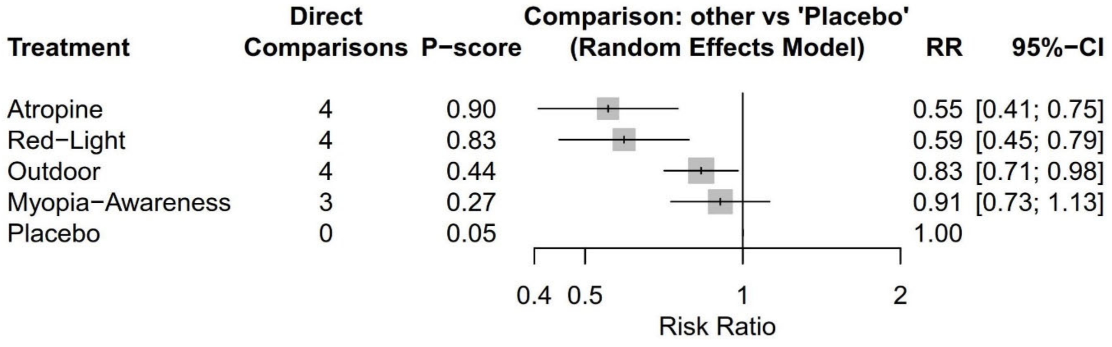

References

1. Høeg KB, Moller F, Jakobsen TM. Stable myopia control during 3-year wear of orthokeratology lenses in Danish children. Invest Ophthalmol Vis Sci. 2024;65(7):6623.
2. X C, M L, J L, M W, X L, C Y, et al. One-year efficacy of myopia control by the defocus distributed multipoint lens: a multicentric randomised controlled trial. The British journal of ophthalmology. 2024.
3. HC C, YT H. The effect of low-concentration atropine combined with auricular acupoint stimulation in myopia control. Complementary therapies in medicine. 2014;22(3):449-55.
4. X C, J X, K C, J E, N B. Soft Contact Lenses with Positive Spherical Aberration for Myopia Control. Optometry and vision science : official publication of the American Academy of Optometry. 2016;93(4):353-66.
5. P C, SW C. Discontinuation of orthokeratology on eyeball elongation (DOEE). Contact lens & anterior eye : the journal of the British Contact Lens Association. 2017;40(2):82-7.
6. KY C, JKW C, GTK W, PH L, SSH C, TC L, et al. Myopia Control Efficacy and Long-Term Safety of a Novel Orthokeratology Lens (MESOK Study)-A Randomized Controlled Clinical Trial Combining Clinical and Tear Proteomics Data. Journal of clinical medicine. 2023;12(9).
7. Drobe B, Spiegel DP, Yang A, Lim EW, Huang Y, China Z, et al. Influence of wearing time on myopia control efficacy of spectacle lenses with aspherical lenslets. Invest Ophthalmol Vis Sci. 2022;63(7):4324-A0029.
8. MH E, RW L, CS L, JK L, BS Y. The Hong Kong progressive lens myopia control study: study design and main findings. Invest Ophthalmol Vis Sci. 2002;43(9):2852-8.
9. M F, S H, R N, K T, H O. Seasonal variation in myopia progression and axial elongation: an evaluation of Japanese children participating in a myopia control trial. Japanese journal of ophthalmology. 2012;56(4):401-6.
10. A H-H, N J, F M, T B, B O, L K. Myopia Control with Low-Dose Atropine in European Children: Six-Month Results from a Randomized, Double-Masked, Placebo-Controlled, Multicenter Study. Journal of personalized medicine. 2023;13(2).
11. He M, Mi B, Zhu Y, Liu L, Zhang Z, Du B, et al. Effects of short-term use of atropine with different concentrations and frequencies on eye safety in children. Chin J Exp Ophthalmol. 2023;41(5):474-82.
12. O H, T H, T F, S I, S H, H T, et al. Assessment of myopic rebound effect after

discontinuation of treatment with 0.01% atropine eye drops in Japanese school-age children. Japanese journal of ophthalmology. 2023;67(5):602-11.
13. XH K, Y Z, Z C, L Z, R H, XQ D, et al. A Randomized Controlled Trial of the Effect of 0.01% Atropine Eye Drops Combined with Auricular Acupoint Stimulation on Myopia Progression. Journal of ophthalmology. 2021;2021:5585441.
14. Lam CS, Tang WC, To CH. Defocus Incorporated Soft Contact (DISC) lens. Acta Ophthalmol. 2015;93.
15. CSY L, WC T, HY Z, PH L, DYY T, H Q, et al. Long-term myopia control effect and safety in children wearing DIMS spectacle lenses for 6 years. Scientific reports. 2023;13(1):5475.
16. D L, JS H, M M, V T, J W, X Z, et al. Control of myopia using diffusion optics spectacle lenses: 4-year results of a multicentre randomised controlled, efficacy and safety study (CYPRESS). BMJ open ophthalmology. 2024;9(1).
17. SS L, G L, M B, PG S, A K, M F, et al. Low-concentration atropine eyedrops for myopia control in a multi-racial cohort of Australian children: A randomised clinical trial. Clinical & experimental ophthalmology. 2022;50(9):1001-12.
18. SC L, MY H, SC H, C C. Soft peripheral contact lens for eye elongation control (SPACE): 1-year results of a double-blinded randomized controlled trial. Contact lens & anterior eye : the journal of the British Contact Lens Association. 2024;47(5):102256.
19. X L, Y H, Z Y, C L, S Z, A Y, et al. Myopia Control Efficacy of Spectacle Lenses With Aspherical Lenslets: Results of a 3-Year Follow-Up Study. American journal of ophthalmology. 2023;253:160-8.
20. Liu Y, Xie L, Guo Y, An T, Yin D, Li Y, et al. Effect of orthokeratology combined with repeated low-level red-light therapy on progressive myopia in adolescents. Recent Advances in Ophthalmology. 2024;44(8):627-31.
21. M D-L, MD P-D. Superdiluted atropine at 0.01% reduces progression in children and adolescents. A 5 year study of safety and effectiveness. Archivos de la Sociedad Espanola de Oftalmologia. 2018;93(4):182-5.
22. Morgan IG, Xiang F, Zeng Y, Mai J, Chen Q, Zhang J, et al. Increased outdoor time reduces incident myopia - The Guangzhou outdoor activity longitudinal study. Invest Ophthalmol Vis Sci. 2014;55(13):1272.
23. M M-C, R R-A, A G-B, FJ M-M, C M-P, MÁ S-T, et al. Five-year results of atropine 0.01% efficacy in the myopia control in a European population. The British journal of ophthalmology. 2024;108(5):715-9.
24. CS N, CW P, EA F, CF L, IB W, J O, et al. A cluster randomised controlled trial

evaluating an incentive-based outdoor physical activity programme to increase outdoor time and prevent myopia in children. Ophthalmic & physiological optics : the journal of the British College of Ophthalmic Opticians (Optometrists). 2014;34(3):362-8.
25. Nkansah EK, Lingham G, Loughman J, Flitcroft I. Biometric treatment efficacy of 0.01% atropine eye drops: analysis of a randomized controlled trial. Invest Ophthalmol Vis Sci. 2023;64(8):1964.
26. EP S, HS C, HC C, TH T. Center-for-Near Extended-Depth-of-Focus Soft Contact Lens for Myopia Control in Children: 1-Year Results of a Randomized Controlled Trial. Ophthalmology and therapy. 2022;11(4):1577-88.
27. WT T, XN L, WJ Z, J L, XY X, HD Z, et al. One-year results for myopia control of orthokeratology with different back optic zone diameters: a randomized trial using a novel multispectral-based topographer. International journal of ophthalmology. 2024;17(2):324-30.
28. JJ W, LA J, DO M, K Z. A randomized trial of the effects of rigid contact lenses on myopia progression. Archives of Ophthalmology. 2004;122(12):1760-6.
29. M X, Z Z, Y J, W W, J Z, R X, et al. Longitudinal Changes in Choroidal Structure Following Repeated Low-Level Red-Light Therapy for Myopia Control: Secondary Analysis of a Randomized Controlled Trial. Asia-Pacific journal of ophthalmology (Philadelphia, Pa). 2023;12(4):377-83.
30. J Z, W F, Y Z, H L, L H, M S, et al. Effects of orthokeratology and spectacle lenses with highly aspherical lenslets on unilateral myopic anisometropia control. Ophthalmic & physiological optics : the journal of the British College of Ophthalmic Opticians (Optometrists). 2024.
31. Zhang HY, Lam CSY, Tang WC, Leung M, Qi H, Lee PH, et al. Myopia control effect of Defocus Incorporated Multiple Segments (DIMS) spectacle lens is influenced by baseline relative peripheral refraction. Invest Ophthalmol Vis Sci. 2022;63(7):254-A0108.
32. H Z, CSY L, WC T, M L, H Q, PH L, et al. Myopia Control Effect Is Influenced by Baseline Relative Peripheral Refraction in Children Wearing Defocus Incorporated Multiple Segments (DIMS) Spectacle Lenses. Journal of clinical medicine. 2022;11(9).
33. HY Z, CSY L, WC T, PH L, DY T, CH T. Changes in relative peripheral refraction in children who switched from single-vision lenses to Defocus Incorporated Multiple Segments lenses. Ophthalmic & physiological optics : the journal of the British College of Ophthalmic Opticians (Optometrists). 2023;43(3):319-26.

34. L T, K C, DL M, LX L, SQ Z, A L, et al. Six-month repeated irradiation of 650 nm low-level red light reduces the risk of myopia in children: a randomized controlled trial. International ophthalmology. 2023;43(10):3549-58.
35. Wong YL, Huang Y, Xue L, Yuan Y, Ye YY, Lim EW, et al. Eye growth pattern of myopic children wearing spectacles lenses with aspherical lenslets compared with non-myopic children. Invest Ophthalmol Vis Sci. 2023;64(8):1425.
36. J C, J W, Z Q, S L, L Z, B Z, et al. Smartwatch Measures of Outdoor Exposure and Myopia in Children. JAMA network open. 2024;7(8):e2424595.
37. X H, J W, Z K, Y Y, X Y, F Z, et al. Pseudomyopia treated with auricular point sticking combined with periocular needle-embedding therapy and prevention of true myopia: a multicenter randomized controlled trial. Zhongguo zhen jiu = Chinese acupuncture & moxibustion. 2024;44(4):405-10.
